# Employment outcomes and experiences of people with seeing disability in Canada: An analysis of the Canadian Survey on Disability 2017

**DOI:** 10.1371/journal.pone.0260160

**Published:** 2021-11-29

**Authors:** Shikha Gupta, Mahadeo Sukhai, Walter Wittich

**Affiliations:** 1 School of Optometry, Université de Montréal, Montréal, Quebec, Canada; 2 Canadian National Institutes for Blind (CNIB), Toronto, Ontario, Canada; 3 Department of Ophthalmology, Queen’s University, Kingston, Ontario, Canada; Northumbria University, UNITED KINGDOM

## Abstract

**Background:**

Many individuals with disabilities face barriers to meaningful employment. Legislation has been put in place to ensure employment equity for individuals with disabilities in Canada. However, little is known about the employment profile and experiences of people with seeing disabilities.

**Objectives:**

The objectives of our research study were to explore the employment rates of people with seeing disabilities in Canada, the factors associated with being employed, and supports and barriers that affect their work participation.

**Methods:**

We used the nationally representative data from the Canadian Survey on Disability (CSD) 2017, collected by Statistics Canada. The CSD is a national cross-sectional survey of Canadians 15 years of age and above who face a functional limitation due to a health-related condition, representing more than 6 million (n = 6,246,640) Canadians. Our analyses focused on people who reported having a seeing disability. A subset of the complete dataset was created, focusing on individuals with a seeing disability. Weighted descriptive analyses were performed using SPSS. Multivariate logistic regression analyses were conducted for individuals between 25–64 years of age to identify predictors of employment.

**Results:**

Out of the estimated 892,220 working-age adults (25–64 years) with a seeing disability who were represented by the survey, 54% were employed, 6% were unemployed and 40% were not in the labour force. Early onset of seeing disability (OR: 1.33; 95% CI: 1.32–1.35), less severe seeing disability (OR: 1.51; 95% CI: 1.49–1.53), education above high school (OR: 2.00; 95% CI: 1.97–2.02) and daily use of the internet (OR: 2.46; 95% CI: 2.41–2.51) were positively related with employment. The top three employment accommodations that were needed and were made available included: modified work hours (45%); work from home (38.5%) and a modified workstation (37%). The top three needed but least available accommodations were technical aids (14%), communication aids (22%) and a computer with specialized software or adaptation (27%). Overall, 26% reported that an accommodation was required but was not made available by the employer. While 75% of individuals with a seeing disability were out of the labour force due to their condition, the remaining identified barriers that prevented them from working which included (top 3): (i) too few jobs available (20%); (ii) inadequate training/experience (19%), (iii) past attempts at finding employment were unsuccessful (19%).

**Conclusion:**

Adults with seeing disability in Canada experience lower labour force participation than the general population. Rigorous programs are required to assist them with the job search, job retraining and workplace accommodations. It is important for governments to improve efforts towards inclusive education and develop strategies that promote digital literacy of employees and job seekers with visual impairments. Although accessibility legislations have been put in place, programs should be established that provide accessibility solutions for various employers, enabling them to hire individuals with different abilities.

## Introduction

Employment is not only a source of income but also a means to identity, independence, participation, health, and social well-being for all individuals. Yet, many individuals living with disabilities worldwide face barriers to meaningful and gainful employment and live under the poverty line [1–3]. Previous research suggests that individuals with disabilities are less likely to find employment than individuals without disabilities, despite similar age and educational attainment [4]. Available estimates from the Canadian Survey on Disability of 2017 indicated that the employment rates (including both full- and part-time employment) for approximately 6 million Canadians aged 15 and over who have one or more disabilities is 59% compared to an 80% employment rate for those without disabilities [5]. Similarly, the Canadian time-use survey indicated that individuals with disabilities spend 60–100 minutes less time in competitive jobs or paid employment while spending more time in household work (18–20 minutes more) per day [6].

Lower employment rates for individuals with disabilities, and the work participation disparity between this population and the “abled” community, have been attributed to several physical, procedural, and attitudinal barriers [7–9]. Some of the commonly reported physical barriers include lack of accessible buildings and workstations, lack of transportation and reliable commuting options, and lack of signage, as well as communication failures [10, 11]. Policies and practices that systematically limit individuals’ work participation are referred to as procedural barriers. They include inaccessible or insensitive hiring processes, lack of workplace accommodations, and lack of transparent communication between employers and disabled employees [12, 13]. The last and probably more concerning barriers to employment for individuals with disabilities are attitudinal barriers. These include assumptions, beliefs, stigma and stereotypes about the person with a disability in general and their ability and potential to perform a particular job [14, 15]. Estimates suggest that between 12% to 51% of workers with disabilities report facing one or more of these barriers, which have deterred them from looking for work or from advancing in their careers [16, 17].

In the case of individuals with visual impairment, the factors that have been tested for associations with employment outcomes in the literature can be divided into three groups: demographic or personal factors, disability-related factors, and service-related factors. Demographic or personal factors include age, sex/gender, education level, aboriginal status, race/ethnicity and self-efficacy and skills. Disability-related factors include age of onset of visual impairment, severity, cause of impairment, and additional disabilities along with sight loss. Lastly, the service-related factors include availability of transportation, availability of mentorship or other vocational rehabilitation services or job training as well as use, availability, and accessibility of assistive devices and technology. Of all these factors, only a few have consistently been identified as significantly associated with employment outcomes. These include education, severity of the seeing disability, the presence of an additional disability, transportation, and accessibility. Effects of other factors, such as age, sex, ethnicity, age of onset of the visual impairment etc. have been inconsistent across several studies [18–20]. Other than these, a few studies indicate that employer attitudes play a critical role in determining employment outcomes of individuals with a visual impairment [21].

Several pieces of legislation have been put in place to ensure employment equity for individuals with disabilities [22]. Specific to Canada, the Employment Equity Act, established in 1995, aimed to achieve workplace equity by ensuring that individuals from designated groups i.e., women, persons with disabilities, indigenous communities, and people of colour are not only treated equally but also provided special accommodations to ensure equal employment opportunities and employment benefits [23]. More recently, the Accessible Canada Act, passed in 2019, focusses on developing and enforcing accessibility standards in Canada, not only within private industry but also within the major sectors that fall under federal jurisdictions, such as transportation, banking, and telecommunications [24]. This new legislation aims to set out the accessibility requirements and enforcement measures to prevent any barriers to employment or overall participation of persons with disabilities.

Despite significant research and policy attention given to employment concerns of people with disabilities, there is not much known about the employment outcomes and experiences of people with seeing disabilities (those who are blind or partially sighted) in Canada [25, 26]. The majority of the empirical evidence (except national surveys) on employment outcomes and experiences of individuals with disabilities is drawn from individuals with physical, mental or learning disabilities, living in the United States or Europe [27]. There are only a few studies that specifically focus on employment among Canadians with sensory disabilities, most of which are either dated or small-scale [28–31]. These estimates are important to ascertain the effectiveness of employment policies (or lack thereof) for people with seeing disabilities [32]. Therefore, the objectives of our research study were to explore:

1. Employment rates of people with seeing disabilities in Canada.
2. Factors associated with being employed for the members of this population.
3. Supports and barriers affecting their work participation.

## Methods

### Canadian survey on disability

To fulfill our study objectives, we used the nationally representative data from the 2017 *Canadian Survey on Disability (CSD)* collected by Statistics Canada [33]. The CSD is a national survey of Canadians 15 years of age and above who experience a functional limitation due to a health-related condition. It is a cross-sectional post-census survey conducted every five-years. The sample for the 2017 CSD was drawn from the pool of individuals who reported facing a long-term health problem or medical condition in the 2016 census. The census and CSD used a two-phase stratified sampling technique, wherein the first phase involved a stratified systematic sample of one in four households occupying a private dwelling in most Canadian regions, and all households in remote areas and on First Nations reserves. The second phase involved the CSD sample which was selected from individuals who reported having difficulty in response to the sub-questions about activities of daily living on the long-form census questionnaire. This sample excluded people living on First Nations reserves and those under the age of 15 as of May 10, 2016.

From the larger pool of census respondents, the CSD used a disability screening questionnaire (DSQ) to identify individuals with one or more of these 10 disability types: hearing, vision, mobility, flexibility, dexterity, pain, learning, mental health, memory, and developmental disabilities. The DSQ was first created and used in the 2012 CSD and was then used in the 2017 CSD with improvements. The DSQ defines disability based on the social model approach and takes a person’s level of functional difficulty and their subjective assessment of the effect of these difficulties on their daily activities into account [33]. Persons who reported a limitation in their day-to-day activities were identified as having a disability and were assigned a disability severity score, based on two criteria. First, based on the intensity of the activity limitations, that measured the degree to which difficulties were experienced across various domains of functioning and, second, based on the frequency of activity limitations, that measured how often daily activities were limited by these difficulties. A final global score was created by adding individual scores on all 10 disability scores.

Along with disability types, the CSD also captured information on specific medical diagnoses that affected daily functioning of individuals, and classified those health conditions according to the 10^th^ International Classification of Diseases (ICD-10) code developed by the World Health Organization [34]. In terms of the scope of information, the CSD included various modules to collect specific information on an individuals’ disability-related characteristics such as the severity and age of onset of the disability; educational attainment and experiences; labour force status and details; access to government services; use of, and unmet needs for health services; use of, and unmet needs for aids and assistive devices; general health status; housebound status; and internet use. All of the information was collected for each of the 10 Canadian provinces and three territories.

The CSD reached a response rate of 69.5% corresponding to 50,000 individuals and representing more than 6 million (n = 6,246,640) Canadians. It is important to note that Statistics Canada suggests each respondent in the sample survey represents not only themselves but also other people who were not sampled. To account for this, each survey respondent was assigned a weight equivalent to the number of people they represented in the larger population, and therefore all estimates provided here are expressed in weighted numbers. More information on sampling and data collection within the CSD is available on the Statistics Canada website and CSD concepts and methods guide [33].

### Study population and analysis

Our analysis focused on people who reported having a seeing disability on the CSD. The ethical clearance for the study was obtained from the institutional review board of *Université de Montréal* (#2020–164 CERC-20-057-D). We also obtained approval from the joint adjudication committee of Statistics Canada to access master files for the CSD data through the Statistics Canada Research Data Centre located at Queen’s University in Kingston, Ontario, Canada. All the data were fully anonymized before access. We created a separate subset of the larger CSD dataset which included individuals who reported having a seeing disability, with or without other types of disabilities. Within the CSD, persons with a seeing disability were identified as persons whose daily activities are limited because of difficulties with their ability to see, and who were identified using two questions on the survey. The first question asked about the level of difficulty a person has in seeing even while wearing their glasses or contact lenses, where applicable. Then, for those with at least some difficulty seeing, a subsequent question asked how often this difficulty limited their daily activities. It is to be noted that persons who reported “some” difficulty seeing but “rarely” being limited in their daily activities, were not identified as having a seeing disability in the CSD.

We analyzed the demographic (i.e., age, sex, province, education) and disability-related characteristics of our study population (i.e., age of onset of disability, severity of disability, number of types of disabilities, number and types of aids or assistive devices used, use of other aids) using descriptive statistics and reported them as counts and proportions. Employment details such as employment status, accommodations, labour force discrimination and barriers to labour force participation were also analyzed using descriptive statistics and reported as percentages. Data that were missing due to non-response or invalid questions were not included in the analysis.

To identify factors that were associated with positive employment outcomes, we built a multivariate binary logistic regression model for working-age adults (25–64 years) with a seeing disability. Two other models were built for males and females separately within this age group [35]. Our main outcome variable for logistic regression was employment divided into two categories–i) employed with either a full-time or part-time job; ii) unemployed and out of the labour force. The independent variables that were included in the logistic models included sex, age of onset of disability, severity of disability, number of types of disabilities, number of aids or assistive devices used, provincial region, education, and frequency of internet use. These factors were chosen because they have previously been associated with employment among individuals with sight loss, blindness, or vision impairment [18–20, 36], and were available in the CSD dataset. All the independent variables that were found to be associated with our outcome variable during bivariate analysis were entered into the model together and were excluded if they were non-significant, using backward elimination. A p-value of < .05 was considered statistically significant. We performed all statistical analyses using the Statistical Package for Social Sciences version 25 (IBM SPSS Statistics, Armonk, New York, USA).

## Results

### Demographic profile

**Fig 1:**
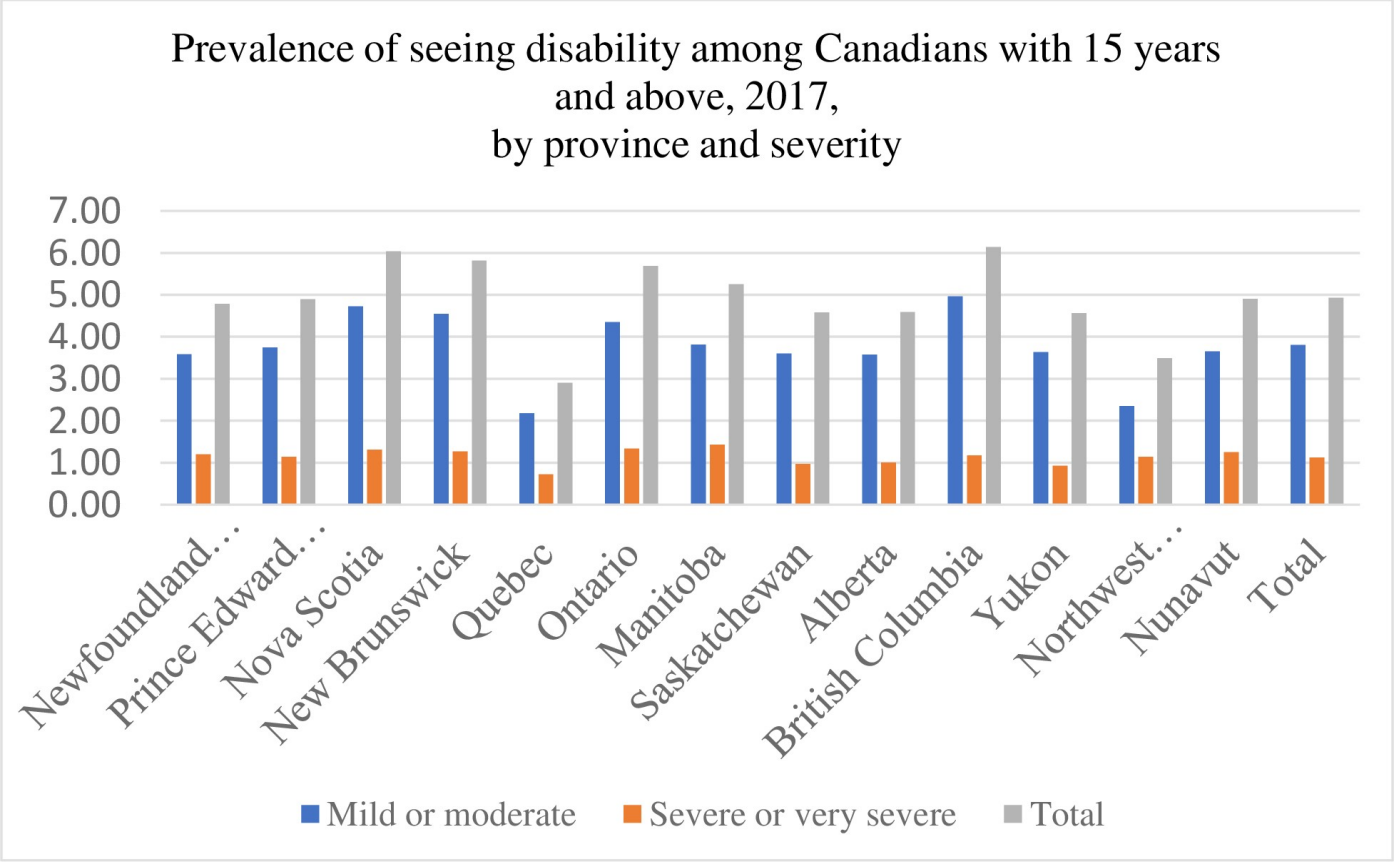
Prevalence of seeing disability across Canadian provinces, 2017.

Of all survey respondents, around 1.5 million (n = 1,519,840) Canadians reported having a seeing disability. These individuals constituted 24% of all people with disabilities in Canada and 5.4% of the total Canadian population (15 years and above). Almost 60% of the individuals with a seeing disability were between 25–64 years of age, 35% were above 65 years and the remaining 5% were between 15 to 24 years of age. The prevalence of a seeing disability was higher among females (60%) compared to males (40%). The province of Ontario had the highest number of individuals living with a seeing disability (44.8%) followed by British Columbia (16.6%) and Quebec (13.5%). In terms of prevalence, British Columbia (6.14%) had the highest prevalence of people with a seeing disability, followed by Nova Scotia (6.04%) (Fig 1).

Out of all individuals with a seeing disability, 77% reported it to be mild to moderate and 23% reported a severe or very severe seeing disability. Approximately 3 in 10 individuals had early onset i.e., between 1 to 15 years of age (25.4%) or at birth (3.8%). Fourteen percent had only a seeing disability, while 27% had two or three types of disabilities and 59% had more than three disabilities. Most individuals with a seeing disability had a high school diploma/certificate or less (45%), followed by 26% with a trade certificate or college diploma (Table 1). More than half (60%) used one or more special aids or devices (other than eyeglasses or contact lenses) to assist in day-to-day functioning. The most used devices were magnifiers (25%), large print reading materials (17%), smartphones (12%) and computers with specialized software (11%).

**Table 1:** Demographic profile of persons with seeing disability in Canada, 2017 (N = 1,519,840).

Population	N (%)
Total individuals with a seeing disability	1519840 (100%)
Age groups	
• 15–24	88010 (5.79%)
• 25–64	892220 (58.70%)
• 65 and above	539610 (35.50%)
Sex	
• Male	616800 (40.58%)
• Female	903040 (59.41%)
Age of onset	
• From birth	57610 (3.79%)
• 1–14 years	385380 (25.35%)
• 15–24 years	145230 (9.55%)
• 25–44 years	265420 (17.46%)
• 45–64 years	397610 (26.16%)
• 65–75 years	71454 (4.70%)
• After 75 years	67710 (4.45%)
• Not known or not stated	129420 (8.51%)
Severity of disability	
• Mild or moderate severe	1173020 (77.18%)
• Severe or very severe	346810 (22.81%)
Number of types of disabilities	
• One	217900 (14.33%)
• Two or three	412540 (27.14%)
• More than three	889400 (58.51%)
Education	
• Less than high school diploma or its equivalent	337320 (22.19%)
• High school diploma or a high school equivalency certificate	344570 (22.67%)
• Trade certificate or diploma	142800 (9.39%)
• College, CEGEP or other non-university certificate/diploma	252910 (16.64%)
• University certificate or diploma below the bachelor’s level	57190 (3.76%)
• Bachelor’s degree	168330 (11.07%)
• University certificate/diploma/degree above bachelor’s level	86970 (5.72%)
• Not known or not stated	129750 (8.53%)
Employment status	
• Employed	560520 (36.88%)
◦ Full time employment	◦ 436080 (77.79%)
◦ Part-time employment	◦ 118020 (21.05%)
• Unemployed	70980 (4.67%)
• Not in the labour force	885800 (58.28%)

### Employment profile and factors affecting employment

In total, 37% of individuals with seeing disabilities over the age of 15 were employed while 58% were out of the labour force and 5% were unemployed in 2017. Of those who reported being employed, 79% were in full-time employment while 21% were in part-time employment. Within the working-age adult cohort (25–64 years) (representing an estimated 892,220 adults with a seeing disability in Canada), 54% were employed, 6% were unemployed and 40% were not in the labour force. Of those who reported being employed, 80% were in full-time employment while 20% were in part-time employment; and 85% had a permanent job while 15% reported having a temporary job. Of the 54% of working-age adults with seeing disabilities (25–64 years) who were employed, the majority (83%) were employed by a third-party employer while 17% were self-employed, which included working for a family business for free.

Bivariate analysis suggested that there was a significant difference in the employment rates between males and females, with males being more likely to be employed than females (OR: 1.36; 95% CI: 1.35–1.37). Higher age, later onset of a seeing disability, greater severity of the seeing disability, use of a seeing aid, use of other general aids, higher number of aids used, and a greater number of types of disabilities were associated with lower employment rates (see Table 2 for strength of these associations). Education above high school and daily use of the internet was positively related with employment. People residing in the Atlantic provinces (New Brunswick, Prince Edward Island, Nova Scotia, and Newfoundland and Labrador) had the lowest employment rates while those living in Northern Canada (Yukon, Northwest territories, and Nunavut) had the highest employment rates (Table 2). Employment rates were reported lowest for those who used a white or identification cane (13%), closed circuit devices (16%) or devices with oversized buttons (22%), and highest for those who used smartphones (41%) or computers with specialized software (41%).

**Table 2:** Employment profile of persons with seeing disability in Canada, 2017 (N = 1,519,840).

	Total		Employed			95% CI	
Age	Numbers	Percent	Numbers	Percent	Odds Ratio*	Lower CI	Upper CI
• 15–24	88010	5.80	43240	49.13	13.04	12.82	13.26
• 25–64	892220	58.80	480070	53.80	15.73	15.55	15.91
• 65 and above	539610	35.56	37200	6.89	Ref.		
Sex							
• Men	616800	40.58	253860	41.15	1.36	1.35	1.37
• Women	903040	59.42	306660	33.95	Ref.		
Age of onset of the seeing disability							
• 0–15 years	442990	29.15	236730	53.43	1.96	1.94	1.98
• 16–44 years	410650	27.02	177260	43.16	1.30	1.28	1.31
• 45–64 years	397610	26.16	146520	36.85	Ref.		
Severity of the seeing disability							
• Mild or moderate	1170700	77.03	460430	39.32	1.58	1.57	1.6
• Severe or very severe	346600	22.80	100480	28.99	Ref.		
Education							
• High school and below	681240	44.82	181680	26.66	Ref.		
• Trade certificate, diploma or college	395590	26.03	189240	47.83	2.52	2.5	2.54
• University degree (Bachelor or above)	312280	20.55	158880	50.87	2.84	2.82	2.87
Number of types of disabilities							
• Has seeing disability only	217900	14.34	122520	56.22	Ref.		
• 2 or 3	412540	27.14	210420	51.00	0.81	0.8	0.82
• more than 3	889400	58.52	227580	25.58	0.27	0.26	0.28
Number of seeing aids used							
• 0 or 1	947450	62.34	411940	43.47	Ref.		
• 2 to 3	424570	27.93	118510	27.91	0.50	0.49	0.51
• 4 to 5	119690	7.87	24900	20.80	0.34	0.33	0.35
• more than 5	25580	1.68	5170	20.21	0.32	0.31	0.34
Total number of aids used							
• 0 or 1	593740	39.06	280650	47.26	Ref.		
• 2 to 3	483980	31.84	159980	33.05	0.55	0.54	0.56
• 4 to 5	244080	16.05	68900	28.22	0.44	0.43	0.45
• more than 5	195500	12.86	50990	26.08	0.39	0.38	0.4
Provincial regions							
• Atlantic provinces	115250	7.58	37730	32.73	Ref.		
• Northern region	3970	0.26	2150	54.15	2.42	2.27	2.58
• Central region	886010	58.29	323800	36.54	1.18	1.16	1.19
• Western region	512070	33.69	196850	38.44	1.28	1.26	1.3
Internet frequency use							
• Never	354970	23.35	26900	7.57	Ref.		
• Daily	900210	59.23	466190	51.78	13.09	12.92	13.27
• Sometimes	261840	17.22	67160	25.64	4.2	4.14	4.27

*Unadjusted odds ratio.

After observing these patterns, we built three multivariate logistic regression models for all working-age adults with seeing disabilities (25–64 years), and for males and females separately within this age group. The following variables were included in the three models: age of onset of a seeing disability, severity of a seeing disability, education, internet use, total number of types of disabilities, total number of aids (seeing and other aids combined), and provincial region. We used the likelihood ratio test, adjusted R square, -2 log likelihood values, the chi-square value, Hosmer and Lemeshow tests, and percentage of correct prediction to assess model robustness.

It is important to note that some of the variables included in the models were highly correlated with each other, such as severity of a seeing disability and number of types of disabilities with number of aids used, as well as education with internet use. When some of these variables were dropped from the model to correct for multicollinearity, we found that model robustness was reduced. As a result, we decided to build a hierarchical logistic model in which group of variables were entered into the model using 4 blocks—block 1: age of onset, severity, number of types of disabilities; block 2: provincial region; block 3: number of aids; and block 4: education, frequency of internet use. The model significance and robustness were highest in this type of modelling and each block of variables contributed significantly to increase model robustness and overall model fit. The separation of variables in blocks reduced some degree of their effect on each other and allowed us to show the combined effect of education and frequency of internet use on employment. It also conveyed that, although a few variables were correlated, they were exerting a significant amount of independent effect on employment, and therefore dropping them out of the model would have reduced its robustness.

We found that all variables that were included in the first model were significantly associated with employment after controlling for each other. Out of all variables, number of types of disabilities had the highest negative effect size, and frequency of internet use had the highest positive effect size in the model. Individuals with a greater number of types of disabilities had lower odds of employment (OR: 0.72; 95% CI: 0.71–0.72) in comparison to those with a seeing disability only. Individuals who used the internet everyday had the highest odds of employment (OR: 2.46; 95% CI: 2.41–2.51) in comparison to those who used the internet sometimes or never. Individuals with university or college degrees had the highest odds of being employed (OR: 2.00; 95% CI: 1.97–2.02) compared with to those with less than high school education. Individuals with a mild or moderate seeing disability, early onset of a seeing disability, those using a lower number of aids or assistive devices and those living in Northern Canadian provinces were significantly more likely to be employed in comparison to those with severe disabilities, late onset of a seeing disability, those using a higher number of aids, and those living in the Central (Ontario and Quebec), Western (Alberta, British Columbia, Saskatchewan and Manitoba) or Atlantic provinces in Canada, after controlling for the other factors mentioned above. Similar trends were found for males-only and females-only models, with the exception of the impact of age of onset. For males, early age of onset of a seeing disability was associated with poorer employment outcomes while, for females, early age of onset of a seeing disability was associated with better employment outcomes (Table 3).

**Table 3:** Logistic regression model for predictors of employment for adults (25–64 years) with seeing disability in Canada, 2017 (N = 892,220).

	Model 1: Both			Model 2: Males only			Model 3: Females only		
		95% confidence intervals			95% confidence intervals			95% confidence intervals	
	Odds Ratio	Lower	Upper	Odds Ratio	Lower	Upper	Odds Ratio	Lower	Upper
Severity of seeing disability (Less vs more severe)	1.51	1.49	1.53	1.20	1.18	1.22	1.84	1.81	1.87
Age of onset between 45–64 years	Ref			Ref			Ref		
Age of onset between 0–15 years	1.33	1.32	1.35	0.84	0.82	0.85	2.21	2.17	2.25
Age of onset between 16–44 years	0.96	0.95	0.97	0.63	0.61	0.64	1.51	1.48	1.54
Number of types of disabilities	0.72	0.71	0.72	0.70	0.70	0.70	0.73	0.72	0.73
Provincial region: Atlantic provinces	Ref			Ref			Ref		
Provincial region: Northern region	2.30	2.07	2.55	2.02	1.74	2.35	2.70	2.33	3.13
Provincial region: Central region	1.14	1.12	1.16	1.41	1.37	1.45	0.98	0.96	1.01
Provincial region: Western region	1.11	1.09	1.13	1.15	1.11	1.18	1.13	1.10	1.16
Number of seeing aids used	0.87	0.87	0.88	0.94	0.94	0.95	0.86	0.85	0.86
Education: High school and below	Ref			Ref			Ref		
Education: Trade certificate, diploma, or college	2.00	1.97	2.02	2.10	2.06	2.14	2.20	2.17	2.24
Education: University degree (Bachelor or above)	1.54	1.52	1.56	1.50	1.47	1.53	1.71	1.68	1.74
Internet Use frequency: Never	Ref			Ref			Ref		
Internet Use frequency: Daily	2.46	2.41	2.51	2.20	2.14	2.26	5.51	5.29	5.75
Internet Use frequency: Sometimes	1.32	1.29	1.35	0.82	0.80	0.85	3.74	3.58	3.90
Constant	1.13			2.28			0.24		
	Model 1 statistics:			Model 2 statistics:			Model 3 statistics		
	-2 Log likelihood: 904312.79			-2 Log likelihood: 384581.91			-2 Log likelihood: 500365.52		
	Model Chi-square: 174295.171			Model Chi-square: 74125.364			Model Chi-square: 116433.37		
	df: 12; p<0.001			df: 12; p<0.001			df: 12; p<0.001		
	% correct prediction: 70.8%			% correct prediction: 73%			% correct prediction: 72.4%		
	Nagelkerke R Square: 0.267			Nagelkerke R Square: 0.265			Nagelkerke R Square: 0.307		

### Employment experiences

To explore employment experiences of individuals with disabilities in Canada, the CSD asked about the reasons that affected the respondents’ ability to change jobs or advance in their career. While 37% of adults with seeing disabilities said that their condition limits the number of hours that can be worked, and adapting to a new work environment would be difficult, more than 25% highlighted stigma or discrimination due to conditions, and difficulty in obtaining required supports or accommodations as their top reasons affecting their ability to advance in their career.

When asked about workplace accommodations, 26% reported that an accommodation was needed but was not made available by the employer. The top three employment accommodations that were needed and were made available included: modified work hours (45%); work from home (38.5%) and modified workstation (37%). The top three needed, but least available accommodations were technical aids (14%), communication aids (22%) and computer with specialized software or adaptation (27%). More than a third of respondents (34%) did not tell their employer about their disability and did not ask for an accommodation (Table 4).

**Table 4:** Workplace accommodations needed and provided to employees with seeing disabilities (25–64 years), 2017 (N = 481,799).

Type of workplace accommodations	Needed		Available	
	numbers	percent	numbers	percent
Modified work hours	171510	35.73	77270	45.05
Modified or different duties	134690	28.06	45170	33.54
Work from home	102420	21.33	39430	38.50
Modified/ergonomic workstation	96280	20.06	35730	37.11
Computer with specialized software or adaptation	56710	11.81	15520	27.37
Human support	32410	6.75	9450	29.16
Technical aids	24170	5.03	3320	13.74
Accessible elevator	23040	4.80	NA	NA
Communication aids	21390	4.46	4740	22.16
Other equipment/help/work arrangements	16450	3.43	10590	64.38
Specialized transportation	13480	2.81	NA	NA
Adapted washrooms	13090	2.73	NA	NA

Notes: NA: These numbers were not available for release due to minimum cell count restrictions.

When asked about labour force discrimination, around 45% adults with seeing disabilities who were employed said that they were disadvantaged in their employment due to their condition. For example, around 14% were refused a job in the past five years, and more than 10% were refused a promotion and a job interview due to their condition. The CSD asked individuals who were unemployed for the reasons for facing difficulty in finding work. The top three reasons that were identified were shortage of jobs (58%); not having enough education or training for available jobs (55%); and not having the work experience required for available jobs (52%). The CSD asked working-age adults who were out of the labour force about barriers that prevented them from working. While 50% of individuals with seeing disabilities identified that they were out of the labour force because of their condition, the remaining identified barriers that prevented them from working which included (top 3): (i) too few jobs available (20%); (ii) inadequate training/experience (19%), (iii) past attempts unsuccessful (19%) (Table 5).

**Table 5:** Barriers to labour force participation among adults with seeing disabilities (25–64 years), 2017 (N = 885,800).

Top barriers to labour force participation	Number	Percent
Few jobs available locally	68050	19.81
Training/experience not adequate	64900	18.89
Past attempts unsuccessful	64810	18.86
Lose additional supports	53490	15.57
Experienced discrimination	49740	14.48
Family responsibilities	40690	11.84
Expected income less than current	39370	11.46
Lack special transportation	32870	9.57
Experience accessibility issues	29700	8.64
Other barriers	18370	5.34
Family/friends discourage	17550	5.10

## Discussion

Our study had three objectives: to present employment rates for people with seeing disabilities in Canada; to identify factors that are associated with being employed for them; and to identify barriers and facilitators that affect employment outcomes of individuals with seeing disabilities, to provide suggestions to improve their labour force participation. The first important finding of our study was that, with all age-groups combined, 37% of individuals with a seeing disability were employed while 58% were out of the labour force and 5% were unemployed in 2017. For working age adults (25–64 years), employment rates were higher as 54% were employed, 6% were unemployed and 40% were not in the labour force. These rates have remained significantly lower than the employment rates for people without disabilities in Canada, which were 74% in 2012 and 80% in 2017 [5, 37].

The employment rates for adults with seeing disabilities in Canada are comparable to the rates from other countries. For example, McDonnall and Sui (2019) utilized the American Community Survey (ACS) data and three other nationally representative surveys in the USA to investigate the employment rates of people with visual impairments (ages 18–64 years) [38]. The authors reported that employment rates have not significantly increased over time, ranging from 36.3% (in 2011) to 44.2% (in 2017) while the unemployment rates ranged from 4% (in 1994–1995) to 19.8% (in 2011). A systematic review by Lund and Cmar (2019) reported the overall employment rates for adults with visual impairment ranged from 32% to 70%, with full-time employment rates between 25% and 46% [18]. Other studies using national survey data from the USA also reported that less than half (43.5%) of working-age adults with visual impairments were employed in 2016, out of which only 29.5% had full-year fulltime employment [39, 40]. In contrast, 76.5% of working-age adults without disabilities were employed [40]. Another study conducted with 559 adults with legal blindness in USA found that 53% or respondents were involved in paid employment, 25% were seeking employment and were unemployed and 20% were not seeking employment [41]. The Canadian National Institute for the Blind (CNIB) Foundation conducted an international survey in 2018 across Canada, Australia, and New Zealand [42]. This study found employment rates among CNIB clients in Canada were 42% (28% full-time, 14% part-time). Australia had an employment rate of 49% and New Zealand had an employment rate of 56% among persons with sight loss. Please note that the definitions for visual impairment, working age, and employment vary across these studies, limiting our ability to make direct comparisons. However, these statistics provide a snapshot of the employment status of individuals with visual impairments across North America, Australia and New Zealand.

With regards to our second objective, we identified a group of modifiable and non-modifiable factors that were associated with positive employment outcomes among adults with seeing disabilities in Canada. Among the non-modifiable factors were severity as well as age of onset of the seeing disability, and the presence of other types of disabilities. Some studies have reported similar findings regarding the impact of the severity of the seeing disability and secondary disabilities on employment [43–45]. These findings identify the potential subgroups such as males with early onset of a seeing disability and individuals with a more severe seeing disability and secondary disabilities who may need additional, rigorous, and targeted services and supports to achieve competitive employment.

Among the modifiable factors that were significantly associated with employment outcomes in our study were higher educational attainment and frequent use of the internet. A large number of previously conducted studies have identified the level of educational attainment as a significant predictor of employment among adults with visual impairments [18, 43, 46, 47]. Despite this evidence, individuals with visual impairments continue to experience barriers in post-secondary education, mostly related to the inaccessibility and lack of assistive technology to access information [48]. These findings emphasize the importance of improving inclusive education efforts for children and youth with seeing disabilities with specific focus on improving access and training on the use of assistive technologies. Our findings also indicate the significance of improving digital literacy among students, employees, and job seekers with visual impairments. The frequent use of the internet and computer-based assistive technology create a positive influence on quality of life of individuals with visual impairments [49]. Further, the skills required to use the internet and technology has also been identified as a relevant factor in vocational training for persons with visual impairments [31].

Another important but unexpected finding of our study was that persons with a seeing disability who used a greater number of aids and assistive devices were less likely to be employed in comparison to those who used none or a smaller number of such aids. Several possible reasons can explain this finding. First, in our stratified analysis, we found that people who used a greater number of aids and assistive devices were more severely visually disabled, which may have resulted in lower employment rates for this subgroup. The second reason may be related to stigma on the part of the potential employer attached to the use of aids and assistive devices, which may negatively affect employability. The use of assistive aids has been shown to result in the negative evaluation of intelligence, achievement, and appearance of people with disabilities by drawing negative attention to the user as an “unordinary person” and a belief that person may be a liability rather than an asset [50, 51]. A recent study found that one of the most common causes of non-acceptance of assistive devices among people with visual impairments was social stigma and the fear of loss of employment, especially in working-age adults [52]. Similarly, another study exploring employment outcomes among adults with Retinitis Pigmentosa found that use of a cane or guide dog significantly reduced the odds of being employed among study participants [53]. These findings point to the fact that social acceptability towards aids and assistive devices is still low, especially for traditional or non-mainstream devices, and efforts are needed to change such attitudes. Involving both disabled and non-disabled communities in the design and development of assistive technologies, personal-contact and friendships and a culture of inclusivity and openness could be some of the strategies to improve the acceptance of assistive devices by both employers and people with visual impairments [54–57].

With regards to our third objective, we found that negative employer attitudes, a lack of assistive technology in the workplace, and disclosure issues affected the ability of workers with seeing disabilities to advance in their careers. Among individuals who were either unemployed or not in the labour force, inadequate training and education and a shortage of jobs were commonly reported barriers to employment. These findings align closely with the findings that are reported in other studies. For example, negative employer attitudes, lack of assistive technology and accommodations, and transportation issues were identified as top barriers in several studies that explored employment outcomes among people with visual impairments [41, 46, 58–60].

Overall, our findings suggest that employment rates of adults with seeing disability in Canada improved slightly between 2012–2017. However, the global pandemic may have caused a huge drop in these rates since 2019 and created a great scope for improving employment rates further. Multipronged strategies are needed that provide appropriate support to job seekers as well as employers. For example, disability diversity and workplace inclusion trainings that highlight success stories can be offered to potential employers to overcome their common inhibitions to hire people living with visual impairments [61]. These efforts may not only improve employment outcomes for people with seeing disabilities but also for people with diverse disabilities. Vocational rehabilitation and job readiness programs and interventions designed for people with visual impairments can focus on providing them with diverse skills sets, such as adaptive internet skills, job application and interview skills, dialogue and negotiation for disability disclosure, independent travel, and networking skills that improve their self-efficacy and chances of success in job search, retention, advancement, and satisfaction. These elements have been previously identified to help those who live with a visual impairment to gain and maintain meaningful careers [59, 60, 62–64].

### Limitations

Our study, as well as the CSD itself, has certain limitations that should be considered while interpreting the study findings. Firstly, the CSD is a cross-sectional survey; hence the cause-effect relationships between different predictors of employment among people with seeing disabilities cannot be determined. Secondly, the survey excluded people living in collective dwellings, on First Nations reserves, and those under the age of 15, and may not be entirely representative. Thirdly, the data collected by the CSD comes from a sample of 50,000 individuals that represent 6 million Canadians, and hence is subjected to sampling and other non-sampling errors (response or measurement bias). Though extensive technical efforts were put in place by Statistics Canada to overcome these limitations including weighting techniques, bootstrapping, and qualitative testing of questionnaire prior to administration. Lastly, our analyses were limited to the variables that were contained in the data set, which restricted our ability to explore this topic beyond the scope of the CSD questionnaire (such as job quality, stigma, or experiences of adults with seeing disability with vocational rehabilitation programs in Canada).

## Conclusion

Adults with a seeing disability in Canada experience lower labour force participation in comparison to the general population. Higher educational attainment and frequent internet use are modifiable factors that improve labour force participation and employment outcomes for adults with visual impairments. This implies that it is important for governments to improve efforts towards inclusive education and develop strategies that promote digital literacy of employees and job seekers with visual impairments. Our findings indicated that the use of a seeing aid and assistive device was associated with lower employment rates which suggests that social acceptability for traditional or non-mainstream devices remains low, and efforts are needed to change such attitudes. Although accessibility legislations have been put in place, programs should be established that provide accessibility solutions to various employers, enabling them to hire individuals with different abilities. Rigorous programs are required that assist individuals with seeing disability with job search, job retraining, disability disclosure, and workplace accommodations.
